# Comparison of the Effectiveness of Vedolizumab and Ustekinumab in Patients with Ulcerative Colitis: A Real-World Retrospective Study

**DOI:** 10.3390/biomedicines12091991

**Published:** 2024-09-02

**Authors:** Kei Nomura, Tomoyoshi Shibuya, Rina Odakura, Mayuko Haraikawa, Hirotaka Ishino, Masayuki Orikasa, Masashi Omori, Masao Koma, Kentaro Ito, Takafumi Maruyama, Osamu Nomura, Dai Ishikawa, Mariko Hojo, Akihito Nagahara

**Affiliations:** 1 Department of Gastroenterology, Juntendo University School of Medicine, 2-1-1 Hongo, Bunkyo-ku, Tokyo 113-8421, Japan; ke-nomura@juntendo.ac.jp (K.N.); mhojo@juntendo.ac.jp (M.H.);; 2 Department of Pathophysiological Research and Therapeutics for Gastrointestinal Disease, Juntendo University School of Medicine, 2-1-1 Hongo, Bunkyo-ku, Tokyo 113-8421, Japan

**Keywords:** ulcerative colitis, ustekinumab, vedolizumab

## Abstract

Ulcerative colitis (UC) is a chronic inflammatory disorder of the large intestine. Data on the comparative effectiveness of biological therapies such as vedolizumab (VDZ) and ustekinumab (UST) remain limited. This retrospective study compared the effectiveness and safety of VDZ and UST in UC patients. Between November 2018 and November 2023, 106 patients were included: 64 received VDZ and 42 received UST. Bio-failure was significantly higher (*p* = 0.005) in the UST group versus the VDZ group. The remission rates at 6, 22, and 54 weeks in VDZ group were 51.6%, 61.3%, and 66.7%. The remission rates at 8, 24, and 56 weeks in the UST group were 66.7%, 65.0%, and 66.7%, respectively. Both treatments were comparable in inducing and maintaining clinical remission over 54–56 weeks, with no significant differences observed in the Lichtiger clinical activity index. Subgroup analyses highlighted the potential short-term effectiveness of UST among cases of bio-failure and a white blood cell level ≥ 9000/µL. Safety profiles were generally favorable, with no significant adverse events. Usutekinumab demonstrated effectiveness as a salvage therapy in patients who failed VDZ. Despite the increased disease severity in the UST group compared to the VDZ group, both groups demonstrated similar remission rates, suggesting UST shows significant efficacy even in moderate to severe UC.

## 1. Introduction

Ulcerative colitis (UC) is a chronic inflammatory condition of the colon marked by episodes of relapse and remission [1]. As a chronic or intermittently recurrent inflammatory condition affecting the gastrointestinal tract, symptoms include bloody diarrhea and abdominal pain. Currently, a definitive cure for UC does not exist, and the cycle of remission and relapse significantly affects the quality of life of affected individuals. Recently, a significant increase has occurred in the number of patients with UC, with considerable progress occurring in the treatment of this disease [2]. A variety of therapeutic agents based on different mechanisms have been developed [3]. Of these, biological drugs have increased in importance in the treatment of UC [4]. This has expanded the management of UC from a clinical response to steroid-free clinical remission, patient-reported outcomes, and mucosal and histological healing [5]. As well as increasing therapeutic alternatives for patients with refractory disease, new treatments may allow personalized patient profiling that optimize their efficacy and safety [6]. However, efficacy, safety, and predictors need to be compared between treatments using head-to-head randomized controlled trials [7].

Real-world studies bridge some gaps in wider safety data and highlight the characteristics of patients excluded from clinical trials [8]. However, real-world data may be retrospective in nature, show inclusion biases, and have missing data. To compare between clinical trials, missing data and inclusion biases need to be allowed for by choosing studies using the appropriate methodology and statistical tools. Patients who are refractory to or cannot tolerate such drugs are usually treated with drugs showing different mechanisms of action. In this instance, the choice for providers and families is between off-label therapies that have a paucity of information concerning their efficacy. Such therapies include vedolizumab (VDZ), which inhibits leukocyte trafficking to the intestinal tract targeting the α4β7 integrin, and ustekinumab (UST), which inhibits interleukin-12 and -23 [9,10,11,12]. The number of reports on the efficacy and safety of VDZ and UST administration have been increasing. This expansion has provided a broader range of treatment choices for patients with moderate to severe cases of UC. While reports on the use of these agents have been increasing annually, comparative studies between the two drugs remain limited. There have been no randomized head-to-head clinical trials comparing VDZ and UST in UC. In a meta-analysis in patients with UC, both drugs induced clinical remission and an endoscopic response [13].

In this study, we aimed to compare the effectiveness and safety of VDZ and UST in patients with UC at our institution in Japan. The real-world data, such as the patients’ background, clinical remission and response rates, non-relapse survival rates, predictive factors of clinical remission, and safety, may contribute to the appropriate clinical management of UC.

## 2. Materials and Methods

### 2.1. Ethical Considerations

This was a retrospective single-center study. The Juntendo University Hospital Ethics Committee approved the study protocol (IRB no. H20-0013, E22-0124). Good clinical practice standards were used, adhering to the principles outlined in the Declaration of Helsinki.

### 2.2. Patients

Between November 2018 and November 2023, 106 patients were enrolled in this study. A UC diagnosis relied on standard criteria involving clinical evaluation, endoscopic examination, and histopathological assessment. Specifically, UC was diagnosed when recurrent bloody stools, inflammation of the colonic mucosa on endoscopic findings, and diffuse inflammatory cell on biopsy histology were observed. Eligible participants were over 16 years of age. The Lichtiger clinical activity index (CAI) ≥5 was used to confirm active UC in patients [14]. They were administered VDZ or UST for the purpose of induction of remission at Juntendo University Hospital. Further exclusion criteria encompassed Crohn’s disease, unclassified colitis, pregnancy, concurrent severe illnesses, and enrollment in alternative clinical trials.

### 2.3. VDZ and UST Treatment

Vedolizumab was administered as induction therapy by intravenous infusions of 300 mg for weeks 0, 2, and 6, and then as a maintenance therapy every 8 weeks. The maintenance therapy dosage remained consistent at 300 mg, identical to the induction dosage [15]. Ustekinumab was administered by intravenous infusions using doses based on body weight (260 mg for body weight <55 kg, 390 mg for ≥55 kg to 85 kg, and 520 mg for ≥85 kg) at week 0 as an induction therapy [11]. This was followed by maintenance therapy, every 8 weeks, of 90 mg of UST according to the judgment of each investigator. No instances occurred where patients needed a reduction in treatment interval or adjustment in dosage.

### 2.4. Data Collection

We used retrospectively collected manual data from Juntendo University Hospital to conduct a retrospective cohort study of UC initiating VDZ or UST therapy. All enrolled patients were monitored in accordance with standard clinical practice protocols. At the time of induction of VDZ and UST, and during each visit, clinical activity was evaluated using CAI. The Mayo endoscopic subscore (MES) classification was used to determine endoscopic severity [16]. Routine laboratory test results were performed, including those for white blood cells (WBCs), hemoglobin, platelets, albumin, C-reactive protein (CRP), and erythrocyte sedimentation rate (ESR). Adverse events that arose during the follow-up period were recorded. Severe adverse events were specifically defined as follows: when treatment was interrupted, hospitalization was required, disability occurred, persistent damage was evident, a colectomy was performed, or mortality ensued. Patients in the VDZ group were seen at each infusion, whereas patients in the UST group were seen for the first infusion and every 2 months thereafter. Information on the concomitant use of 5-aminosalicylic acid, immunomodulators, including thiopurines (azathioprine and mercaptopurine), and steroids was recorded at inclusion and during all follow-up visits.

### 2.5. Clinical Evaluation

For clinical activity assessment, we utilized CAI. A higher score on the scale indicated severer disease, with a score range from 0 to 21. Clinical remission was defined as CAI ≤ 4, and a clinical response was defined as a reduction in CAI by ≥3 points with CAI ≤ 10. Maintained efficacy was defined as the absence of CAI exacerbation and a necessity for intensifying treatments. Treatment evaluations were compared at specific time points: 6 weeks after VDZ induction and 8 weeks after UST induction (early-term assessment); 22 weeks after VDZ induction and 24 weeks after UST induction (intermediate-term assessment); and 54 weeks after VDZ induction and 56 weeks after UST induction (long-term assessment).

### 2.6. Subgroup Analysis

Subgroup analyses aimed to assess differences in remission to VDZ and UST among the following subgroups: male, age, duration, extensive, CAI, MES, bio-naïve, WBCs, CRP, neutrophil/lymphocyte ratio (NLR), and lymphocyte/monocyte ratio (LMR). Subgroups were analyzed to compare clinical outcomes between VDZ and UST groups. Clinical remission rates were evaluated at 6–8 and 54–56 weeks of treatment in a subgroup analysis.

### 2.7. Statistical Analysis

We conducted a retrospective analysis. GraphPad Software Version 10.1.1 was used to analyze data. The mean and standard deviation were calculated for age, clinical findings, and duration of disease. Proportions of the population were determined for sex and disease location. The statistical significance in differences in pairwise comparisons were determined using a Mann–Whitney U test, Fisher’s exact test, or Pearson’s chi-square test. The Kaplan–Meier method was used to assess non-relapse survival. Statistical significance was considered at *p* ≤ 0.05.

## 3. Results

### 3.1. Patient Characteristics

Our cohort included 64 patients treated with VDZ and 42 patients treated with UST. Table 1 outlines the clinical characteristics of patients with UC. On diagnosis, most patients presented with moderate to severe symptoms. In comparing the two groups, a statistically significant difference was noted for a history of biologics use (*p* = 0.005). However, no significant difference was observed in a history of the use of anti-tumor necrosis factor (TNF) inhibitor between the two groups. Laboratory data at the time of initiation of biological therapy were compared between the two groups; WBCs, hemoglobin, platelets, albumin, CRP, and ESR were assessed. It was revealed that WBC and platelet levels and ESR were significantly higher in patients of the UST group compared to those in the VDZ group (*p* = 0.04, 0.01, and 0.03, respectively). Additionally, it was found that the albumin level was significantly lower in patients of the UST group compared to those of the VDZ group (*p* = 0.02). No significant differences were observed in the other parameters, including CAI and MES. Based on the above results, it was shown that UC in patients of the UST group exhibited a more severe tendency compared to those in the VDZ group.

### 3.2. Comparisons of Effectiveness between VDZ and UST

**Figure 1:**
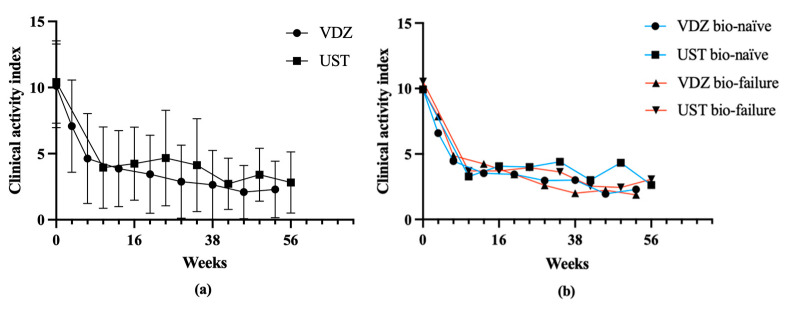
Change in Lichtiger clinical activity index (CAI) after induction of vedolizumab (VDZ) and ustekinumab (UST) (**a**). Change of CAI in bio-naïve and bio-failure groups (**b**).

Overall changes in CAI were observed for patients in both the VDZ and UST groups. Significant decreases were noted during the early-, intermediate-, and long-term periods from induction. However, no significant differences were observed between the VDZ and UST groups during the early-, intermediate-, and long-term periods. When comparing bio-naïve and bio-failure groups, both groups displayed decreases in CAI. Comparisons of the mean CAI between VDZ and UST groups for bio-naïve, as well as for bio-failure, did not reveal any significant differences (Figure 1, Table 2).

### 3.3. Clinical Remission and Response Rates with VDZ and UST

**Figure 2:**
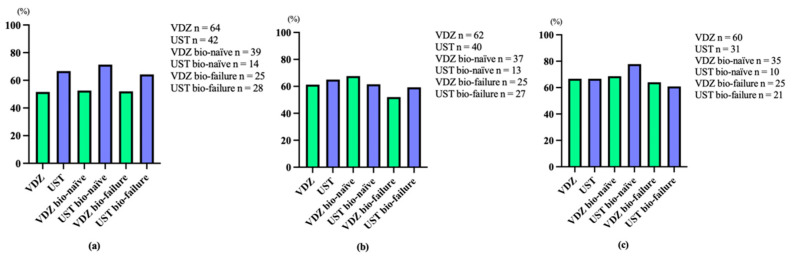
Clinical remission rates with vedolizumab (VDZ) and ustekinumab (UST) at weeks 6 and 8 (**a**), at weeks 22 and 24 (**b**), and at weeks 54 and 56 (**c**).

**Figure 3:**
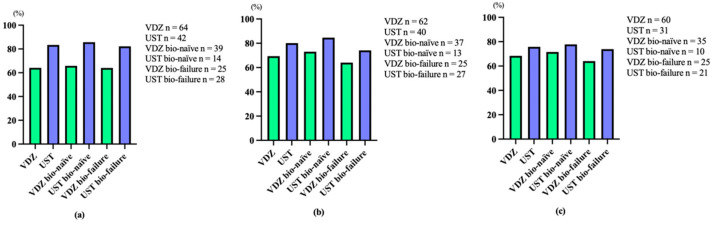
Clinical response rates with vedolizumab (VDZ) and ustekinumab (UST) at weeks 6 and 8 (**a**), at weeks 22 and 24 (**b**), and at weeks 54 and 56 (**c**).

Additionally, overall clinical remission rates at 6, 22, and 54 weeks after VDZ administration were 51.6% (bio-naïve 52.6%, bio-failure 52.0%), 61.3% (bio-naïve 67.6%, bio-failure 52.0%), and 66.7% (bio-naïve 68.6%, bio-failure 64.0%), respectively. Overall clinical remission rates at 8, 24, and 56 weeks after the administration of UST were 66.7% (bio-naïve 71.4%, bio-failure 64.3%), 65.0% (bio-naïve 61.5%, bio-failure 59.3%), and 66.7% (bio-naïve 77.8%, bio-failure 60.9%), respectively (Figure 2). Furthermore, overall clinical response rates at 6, 22, and 54 weeks after the administration of VDZ were 64.1% (bio-naïve 65.8%, bio-failure 64.0%), 69.4% (bio-naïve 73.0%, bio-failure 64.0%), and 68.4% (bio-naïve 71.5%, bio-failure 64.0%), respectively. Overall clinical response rates at 8, 24, and 56 weeks after UST administration were 83.4% (bio-naïve 85.7%, bio-failure 82.2%), 80.0% (bio-naïve 84.6%, bio-failure 74.1%), and 75.8% (bio-naïve 77.8%, bio-failure 73.9%), respectively (Figure 3). No significant differences were noted between VDZ and UST groups in terms of remission and response rates. In addition, no significant difference was found between bio-naïve and bio-failure groups.

### 3.4. Non-Relapse Rates with VDZ and UST

**Figure 4:**
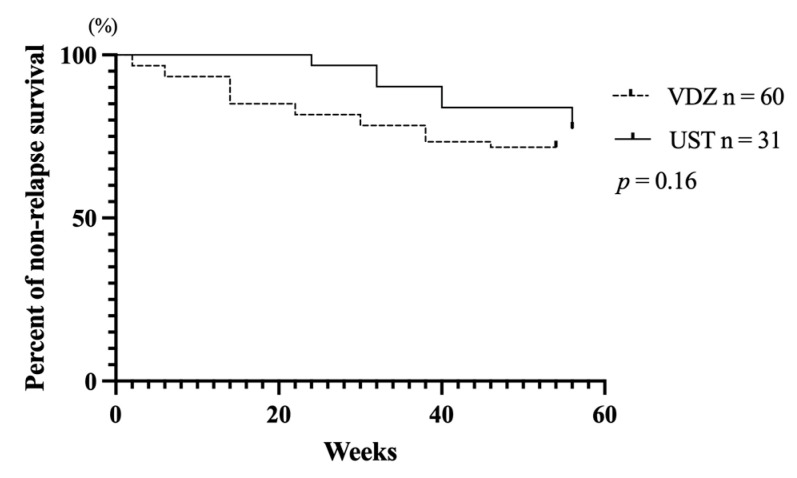
Kaplan–Meier plots for relapse-free survival of patients treated with vedolizumab (VDZ) and ustekinumab (UST) up to 54 and 56 weeks after administration.

The survival curve depicted the cumulative non-relapse rate up to 54 weeks after VDZ administration and 56 weeks after UST administration. This rate represents the percentage of cases that did not require surgery or medication changes due to worsening symptoms. No difference was observed between VDZ and UST groups (*p* = 0.16; Figure 4).

### 3.5. Effectiveness of UST in VDZ Failure

Within the UST group, a notable trend was observed where 23 out of 28 cases in bio-failure had previously failed VDZ, indicating a higher proportion of VDZ failure. In 16 cases of VDZ failure, the first biological agent was VDZ, followed by UST as the second agent. Furthermore, comparing the remission and response rates of UST between bio-naïve cases and those in VDZ failure as the first biologics cases, no significant differences were observed in any of the early-, intermediate-, and long-term assessments, indicating favorable outcomes across all periods (Table 3). Although the remission rate of the VDZ-failure group was slightly lower compared to that of the bio-naïve group, >50% of patients achieved remission at all time points.

### 3.6. Subgroup Analysis

**Figure 5:**
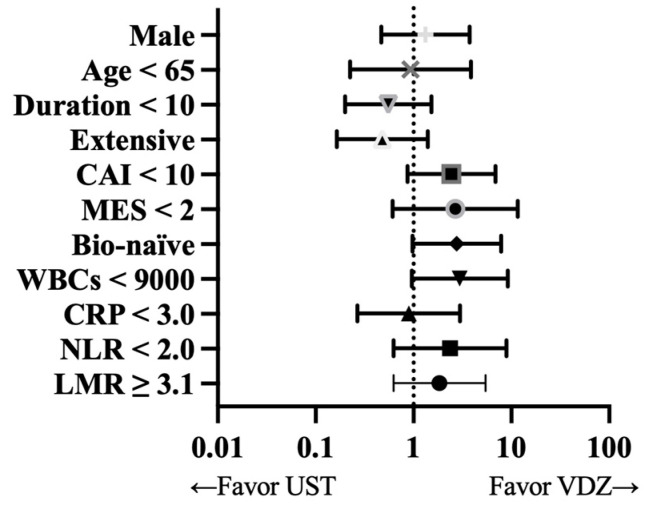
Forest plot for clinical remission in vedolizumab (VDZ) and ustekinumab (UST) groups at early term. CAI: Lichtiger clinical activity index; CRP: C-reactive protein; LMR: lymphocyte/monocyte ratio; MES: Mayo endoscopic subscore; NLR: neutrophil/lymphocyte ratio; WBCs: white blood cells.

In a subgroup analysis, VDZ and UST showed similar clinical remission rates in all subgroups at 6–8 weeks of treatment (Figure 5). However, higher rates of clinical remission were observed in the VDZ group in the case of bio-naïve patients (*p* = 0.053) and a WBC level <9000/µL (*p* = 0.054). The subgroup analyses suggested a potential advantage of UST with regard to short-term effectiveness among cases of bio-failure and a WBC level ≥9000 /µL. Vedolizumab and UST showed similar clinical remission rates in all subgroups at 54–56 weeks of treatment; no significance difference was observed.

### 3.7. Safety

During treatment, a case of pneumonia was noted in the VDZ group, but it was found not to be related to VDZ. Adverse events were not observed up to 56 weeks after the start of UST in this study.

## 4. Discussion

In this Japanese study, we describe how VDZ and UST induced and maintained clinical remission at 54 to 56 weeks of treatment in patients with UC. It was shown that VDZ and UST were both effective in the early-, intermediate-, and long-term periods. Subgroup analyses suggested a potential advantage of UST in short-term effectiveness among cases of bio-failure and a WBC level ≥9000 /µL.

Significant differences were observed in patient backgrounds between VDZ and UST groups. Specifically, the UST group showed a higher proportion of cases with previous VDZ failure, indicating a potential selection bias toward more treatment-resistant patients. Although no significant differences were found in CAI and MES, significant differences were found in WBC, platelet, and albumin levels and in ESR, with a higher severity level observed in the UST group compared to the VDZ group. Both VDZ and UST groups demonstrated the effective induction and maintenance of remission in both bio-naïve and bio-failure cases across various timeframes from early- to long-term periods. These results suggest that both agents could be viable treatment options for patients with UC, regardless of their prior treatment history. Additionally, based on the patient characteristics, it was shown that UC in the patients of the UST group exhibited a more severe tendency compared to those in the VDZ group. Despite greater severity in the UST group compared to the VDZ group, both VDZ and UST demonstrated similar remission rates, suggesting that UST demonstrated significant efficacy even in cases with moderately severe symptoms.

In a recent network meta-analysis, UST and VDZ were compared as induction and maintenance therapies in bio-naïve and anti-TNF-exposed patients with UC. Differences were found in most clinical outcomes between VDZ and UST. Ustekinumab had a higher propensity to induce clinical remission than VDZ in anti-TNF-exposed patients, while a trend was noted in favor of VDZ in bio-naïve patients [17]. Conversely, we did not find any difference between these two drugs at 6–8, 22–24, and 54–56 weeks of treatment with regard to effectiveness. Notably, the vast majority of patients included in the present study had been previously exposed to at least one biological agent before inclusion.

Also, the survival analysis comparing the cumulative non-relapse rates between VDZ and UST treatments revealed no statistically significant difference up to 54 weeks post-VDZ administration and 56 weeks post-UST administration. This finding suggests that both VDZ and UST are comparably effective in maintaining clinical stability without necessitating surgical intervention or changes in medication due to symptom exacerbation within the observed timeframe. The absence of a significant difference in outcomes between the two groups indicates that either therapy could be a viable option for patients requiring sustained disease control. Further research with a larger sample size or extended follow-up may be necessary to identify any long-term distinctions in efficacy between these treatment modalities.

Furthermore, the effectiveness of UST in cases of prior VDZ failure indicated its potential as a salvage therapy option for patients who did not respond adequately to VDZ. The reason for the higher number of UST cases of prior VDZ failure is likely because VDZ was approved earlier than UST for UC, owing to the former’s lower systemic immune suppression [18].

In the subgroup study, the benefit of VDZ compared to UST was less pronounced in patients who were not bio-naïve and had a WBC level <9000 /µL in the short term. The results suggested a potential advantage for UST with regard to short-term effectiveness among cases of bio-failure and a WBC level ≥9000 /µL. In other studies, there was no significant difference in therapeutic effects between VDZ and UST, consistent with the findings of this study [19,20,21]. Two reports showed that VDZ and UST were similarly effective in inducing clinical remission in the failure of anti-TNF. However, a report showed that UST is more effective than VDZ in inducing endoscopic and histological remission at week 16 after the failure of anti-TNF in UC. This comparison with the literature emphasizes the need for further research to clarify the conditions under which UST might outperform VDZ, especially in achieving different types of remission in UC. It also underscores the complexity of treatment decisions in bio-failure cases, where individual patient factors and disease characteristics must be carefully considered.

No clinically relevant differences were observed between the two drugs with respect to safety. However, it is difficult to reach a conclusion since our follow-up was relatively short. Both VDZ and UST are considered as associated with favorable safety profiles even in susceptible populations. Multiple studies have reported a comparable safety profile for both drugs in patients with UC, indicating no significant differences between them. These results were consistent with the long-term extension of real-world studies of VDZ and UST [13,19,20,21].

A limitation of this study is that our analysis was conducted at a single facility, resulting in a restricted number of cases. An additional limitation of our study is the sample size discrepancy between the two groups, which may reduce the accuracy of results. Additionally, this study is limited by the fewer cases where patients transitioned from UST to VDZ compared to from VDZ to UST, thereby rendering its investigation incomplete. Also, this study was retrospective in nature and recruited a limited number of cases with diverse treatment histories. For example, patients in our study showed higher rates of usage of immunomodulators in VDZ and UST groups (39.1% and 31.0%, respectively) compared with other studies. Additionally, the quality of data needs to be improved so that studies using real-world data can more closely emulate randomized clinical trials. This study yields preliminary data on a comparison of VDZ and UST in patients with UC. However, prospective, randomized clinical trials are needed to validate our findings because these data were obtained from the retrospective single-center study. Including a cost-effectiveness analysis in this study could provide valuable insights for healthcare providers and policymakers, helping them to make informed decisions about the economic implications of choosing between VDZ and UST for UC treatment. However, since this study is retrospective and primarily focused on clinical outcomes, it did not include the detailed cost data required for a comprehensive cost-effectiveness analysis. Analyzing these costs in relation to the clinical outcomes would provide a clearer understanding of the economic value of each therapy, aiding in more cost-effective decision making in clinical practice.

## 5. Conclusions

In conclusion, VDZ and UST were similarly effective for inducing clinical remission in patients with UC. Both VDZ and UST demonstrated sufficient efficacy in inducing remission and maintaining long-term remission not only in bio-naïve patients but also in those showing bio-failure. It was revealed that the history of biologics use, WBC and platelet levels, and ESR were significantly higher in patients of the UST group compared to those in the VDZ group. Despite the more severe disease in patients treated with UST compared to VDZ, both drugs demonstrated similar remission rates, suggesting that UST has significant efficacy even in cases with moderate to severe symptoms. Additionally, UST demonstrated effectiveness as a salvage therapy in patients who failed VDZ. These findings highlight the potential of both VDZ and UST as effective treatment options for patients with UC, regardless of their treatment history.

## Figures and Tables

**Table 1:** Characteristics of patients with ulcerative colitis.

Characteristics	Vedolizumab, n = 64	Ustekinumab, n = 42	*p* Value
Age (years), mean ± SD [range]	48.0 ± 15.1 [20–81]	45.0 ± 16.4 [19–80]	0.08
Male/Female, n (%)	35/29 (54.7/45.3)	20/22 (47.6/52.4)	0.48
Duration of disease (years), mean ± SD [range]	10.5 ± 9.4 [0–38]	9.0 ± 10.0 [1–37]	0.34
Disease location			
Proctitis, n (%)	5 (7.8)	2 (4.8)	
Left-sided colitis, n (%)	19 (29.7)	9 (21.4)	
Extensive colitis, n (%)	40 (62.5)	31 (73.8)	
CAI, mean ± SD [range]	10.1 ± 3.2 [5–18]	10.4 ± 3.1 [5–16]	0.54
MES, mean ± SD [range]	2.1 ± 0.8 [0–3]	2.3 ± 0.7 [1–3]	0.19
Previous medications			
Bio-naïve, n (%)	39 (60.9)	14 (33.3)	0.005
Biologics	25 (39.1)	28 (66.7)	0.005
One line, n (%)	17 (26.6)	19 (45.2)	
Two lines, n (%)	6 (9.4)	8 (19.1)	
Three lines, n (%)	2 (3.1)	1 (2.4)	
Anti-TNF, n (%)	25 (39.1)	12 (28.6)	0.27
Tofacitinib, n (%)	0	0	
Vedolizumab, n (%)		23	
Ustekinumab, n (%)	1		
Co-treatments			
5-aminosalicylic acid, n (%)	54 (84.4)	36 (85.7)	0.85
Steroids, n (%)	16 (25.0)	9 (21.4)	0.67
Thiopurines, n (%)	25 (39.1)	13 (31.0)	0.39
WBCs (/µL), mean ± SD [range]	7200 ± 3070 [3300–21,700]	8500 ± 3086 [2800–19,200]	0.04
Hemoglobin (g/dL), mean ± SD [range]	12.7 ± 2.2 [7.7–17.6]	11.6 ± 2.0 [6.8–15.5]	0.08
Platelets (×10^4^/µL), mean ± SD [range]	29.8 ± 10.5 [13.5–75.0]	37.3 ± 14.9 [13.9–93.8]	0.01
Albumin (g/dL), mean ± SD [range]	3.9 ± 0.8 [1.3–4.8]	3.4 ± 0.7 [1.9–4.4]	0.02
CRP (mg/dL), mean ± SD [range]	0.38 ± 4.88 [0.02–33.20]	0.99 ± 5.15 [0.03–29.48]	0.35
ESR (mm), mean ± SD [range]	25 ± 25 [2–100]	35 ± 29 [2–129]	0.03

CAI: Lichtiger clinical activity index; CRP: C-reactive protein; ESR: erythrocyte sedimentation rate; MES: Mayo endoscopic subscore; SD: standard deviation; TNF: tumor necrosis factor; WBCs: white blood cells.

**Table 2:** Comparisons of Lichtiger clinical activity index for vedolizumab and ustekinumab.

	Vedolizumab	Ustekinumab	*p* Value
Induction	10.1 ± 3.2 [5–18]	10.4 ± 3.1 [5–16]	0.63
W6, W8	4.5 ± 3.4 [0–13]	3.9 ± 3.1 [0–14]	0.50
W22, W24	3.4 ± 3.0 [0–13]	4.7 ± 3.6 [0–14]	0.15
W54, W56	2.3 ± 2.1 [0–9]	2.8 ± 2.3 [1–10]	0.39
Bio-naïve			
Induction	9.9 ± 3.0 [5–17]	9.9 ± 2.7 [6–15]	0.98
W6, W8	4.5 ± 3.3 [0–14]	3.3 ± 2.4 [0–9]	0.33
W22, W24	2.9 ± 2.8 [0–13]	4.0 ± 2.4 [0–8]	0.14
W54, W56	2.1 ± 2.0 [0–9]	2.6 ± 1.0 [1–4]	0.25
Bio-failure			
Induction	10.0 ± 3.2 [6–17]	10.5 ± 3.3 [5–16]	0.58
W6, W8	4.9 ± 3.4 [0–13]	3.7 ± 3.1 [0–13]	0.20
W22, W24	3.4 ± 2.5 [0–9]	4.0 ± 3.8 [0–14]	0.96
W54, W56	1.9 ± 1.8 [0–7]	3.1 ± 2.9 [0–10]	0.29

**Table 3:** Comparisons of effectiveness of ustekinumab between bio-naïve and vedolizumab failure groups.

	Bio-Naïve, n = 14	Vedolizumab Failure, n = 16	*p* Value
Remission, Response			
W8, n (%)	10/14 (71.4), 12/14 (85.7)	11/16 (68.8), 13/16 (81.3)	0.87, 0.74
W24, n (%)	8/13 (61.5), 11/13 (84.6)	9/15 (60.0), 11/15 (73.3)	0.93, 0.47
W56, n (%)	8/10 (77.8), 8/10 (77.8)	7/12 (58.3), 8/12 (66.6)	0.27, 0.48

## Data Availability

The original contributions presented in this study are included in the article. Further inquiries can be directed to the corresponding author.
